# Utilizing molecular docking and cell validation to explore the potential mechanisms of lupenone attenuating the inflammatory response via NF‐κB pathway

**DOI:** 10.1038/s41598-024-51150-3

**Published:** 2024-01-05

**Authors:** Xiangpei Wang, Mei Liu, Xiaofen Li, Mei Zhang, Feng Xu, Hongyun Liu, Hongmei Wu

**Affiliations:** 1 School of Chinese Ethnic Medicine, Guizhou Minzu University, Guiyang City, 550025 Guizhou People’s Republic of China; 2 Department of Pharmacy, Guizhou University of Traditional Chinese Medicine, Guiyang City, 550025 Guizhou People’s Republic of China; 3 School of Physical and Mathematical Sciences, Nanyang Technological University, Singapore, 637371 Singapore

**Keywords:** Cell biology, Computational biology and bioinformatics

## Abstract

Diabetic nephropathy (DN), a common microvascular complicating disease of diabetes. Lupenone, a pentacyclic triterpenoid, has anti-inflammatory effects and can prevent type 2 diabetes mellitus and treat renal damage, however, the effects and mechanisms of lupenone in DN remain unclear. Thereby,the MTT method was used to investigate the antiproliferative effect of lupenoneon the cell line rat glomerular mesangial cells (HBZY-1). Molecular docking was used to investigate the combination of lupenone and MCP-1, IL-1β, TNF-α, IKKβ, IκBα, and NF-κB p65 proteins. The expression of mRNA of the pro-inflammatory cytokines (MCP-1, IL-1β and TNF-α) and the NF-κB signalling pathway in HBZY-1 cells were assessed by RT-PCR. The protein expressions of pro-inflammatory cytokines and NF-κB pathway were got by Western blot. Result showed that lupenone inhibited the proliferative activity of HBZY-1 cells at non-cytotoxic concentrations. Molecular docking results showed that lupenone combined well with the target proteins. Moreover, lupenone could significantly reduced the mRNA and protein expressions for pro-inflammatory cytokines and IKKβ, p-p65 and p-IκBα. Lupenone may play an anti-inflammatory role in DN treatment by inhibiting the NF-κB signalling pathway. These results provided a new understanding of the pharmacological mechanisms of lupenone in treatment of DN.

## Introduction

Diabetic nephropathy (DN) is a chronic disease affecting the structure and function of the kidneys, caused by diabetes mellitu. DN is one of the most common microvascular complications of diabetes mellitus and a major cause of end-stage renal disease^1–3^. Mesangial cell hyperplasia, mesangial cell hypertrophy and extracellular matrix (ECM) accumulation are the main features of DN. The typical clinical manifestations of DN include massive proteinuria, hyperglycaemia, hypertension, and oedema. It is widely believed that the combined effect of environmental and genetic factors may be a contributing factor in DN^4–6^. In addition, research has shown that high blood sugar is the main driving force for the occurrence and development of DN, and high blood sugar and its secondary products can activate nuclear factors- κB (NF-κB) inflammatory pathways^7^. Various pro-inflammatory cytokines are involved in the pathogenesis of DN and they accelerate inflammation^8,9^. In recent years, the prevalence of DN has also increased rapidly^10^, seriously affecting the prognosis of patients and resulting in a huge financial burden. Therefore, there is an urgent need for research into new drugs or therapeutic approaches to treat the early inflammatory state of DN.

Lupenone, a pentacyclic triterpenoid compound, is isolated from the roots of *Musa basjoo* Sied.et Zucc, bananas, including the tropical fruit banana (*Musa nana* Lour), and emperor banana (*Musa acuminata cv.* Mas (AA)), and various fruit peels^11–13^. It is also found in the *Platycodon grandiflorum* plant, *Salvia miltiorrhiza*, *Adenophora tetraphylla* (Thunb.) Fisch.^14^, and legume chicken blood vine (*Spatholobus suberectus* Dunn) ^15^. Our previous research confirmed that lupenone has anti-inflammatory effects^16,17^ and prevents and treats type 2 diabetes and type 2 DN^18–20^. High-glucose stimulation of glomerular mesangial cells is a commonly used in vitro cell model to study DN pathogenesis^21^. Increased mesangial cell proliferation in response to high glucose stimulation leads to ECM accumulation and also leads to the development of an inflammatory response^22,23^. ECM accumulation is a typical characteristic of DN. In addition, high-glucose stimulation increases the release of pro-inflammatory factors such as TNF-α and IL-1β and activates of the NF-κB signalling pathway in rat glomerular mesangial cells (HBZY-1)^24,25^. However, precisely how lupenone modulates the inflammatory response in DN and its targets remain unclear.

Therefore, in this study, we aimed to verify the effect of lupenone on MCP-1, IL-1β, TNF-α, IKKβ, IκBα, and NF-κB p65 using molecular docking. Moreover, an in vitro model using HBZY-1 cells induced by high sugar was established to estimate the regulatory effect of lupenone on inflammation and proliferation of HBZY-1 cells under high-glucose conditions.

## Results

### Lupenone suppresses high‐glucose-induced HBZY-1 cell proliferation

**Figure 1:**
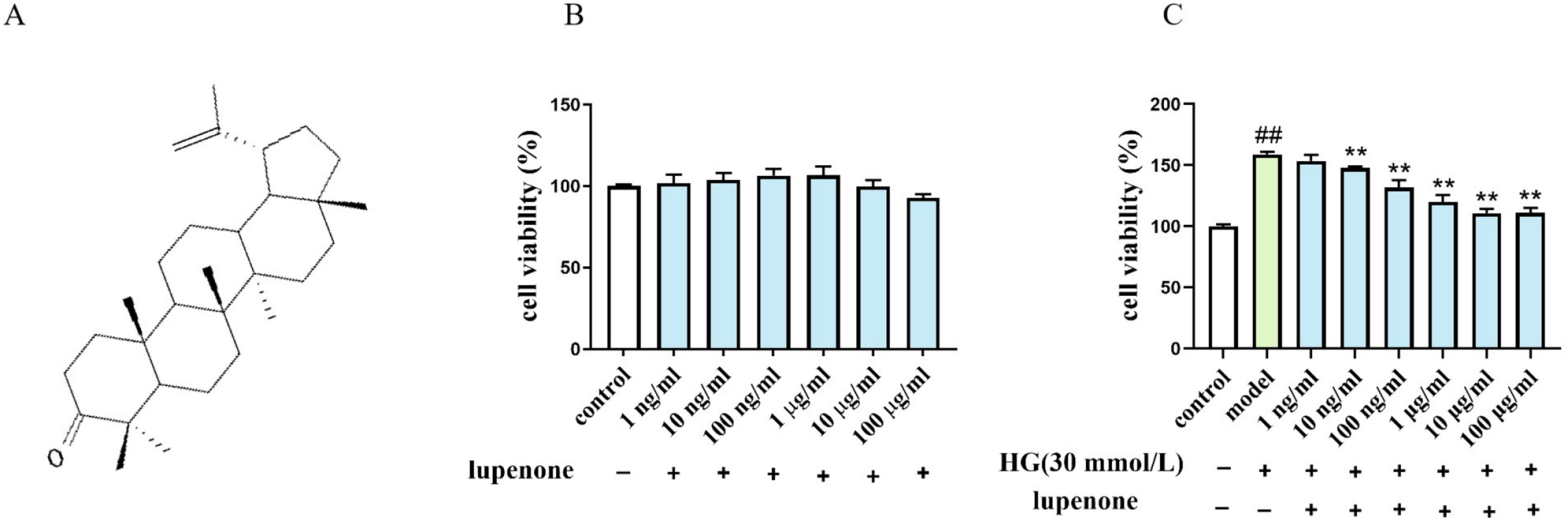
(**A**) Chemical structure of lupenone, (**B**) HBZY-1 cell viability after treatment with lupenone (0 ng/mL-100 μg/mL) for 24 h, (**C**) Inhibition of cell proliferation by lupenone (0 ng/mL–100 μg/mL) for 24 h in HBZY-1 cells stimulated with 30 mmol/L glucose (except for the control). Results are presented as mean ± SD. Significance: ^##^*P* < 0.01 versus the control, **P < 0.01 versus the model.

Our results showed that lupenone was not toxic (*P* > 0.05) to HBZY-1 cells in the range of 1 ng/mL-100 μg/mL (Fig. 1B). High-glucose challenge led to a noticeable increase in HBZY-1 cell multiplication compared to the control (Fig. 1C). Moreover, stimulation of HBZY-1 cells with different concentrations of lupenone (1 ng/mL-100 μg/mL) showed that lupenone at concentrations of 10 ng/mL–10 μg/mL significantly inhibited high glucose-induced proliferation of HBZY-1 cells in a dose-dependent manner (*P* < 0.01). Furthermore, the inhibition rate was 7.07% in the 10 ng/mL dose group and 30.11% in the 100 μg/mL dose group. It is worth noting that the inhibitory effect of lupenone on high glucose-induced proliferation of HBZY-1 cells was lower in the 100 μg/mL dose group than that in the 10 ug/ml dose group (Table 1).

**Table 1:** Cell inhibition.

Group	Inhibition (%)
control	–
model	–
1 ng/ml lupenone	3.39
10 ng/ml lupenone	7.07
100 ng/ml lupenone	17.13
1 μg/ml lupenone	24.52
10 μg/ml lupenone	30.27
100 μg/ml lupenone	30.11

Control: cells were incubated without glucose medium. Model: cells were incubated with 30 mmol/L glucose medium. Lupenone treatments.

### Docking results

**Figure 2:**
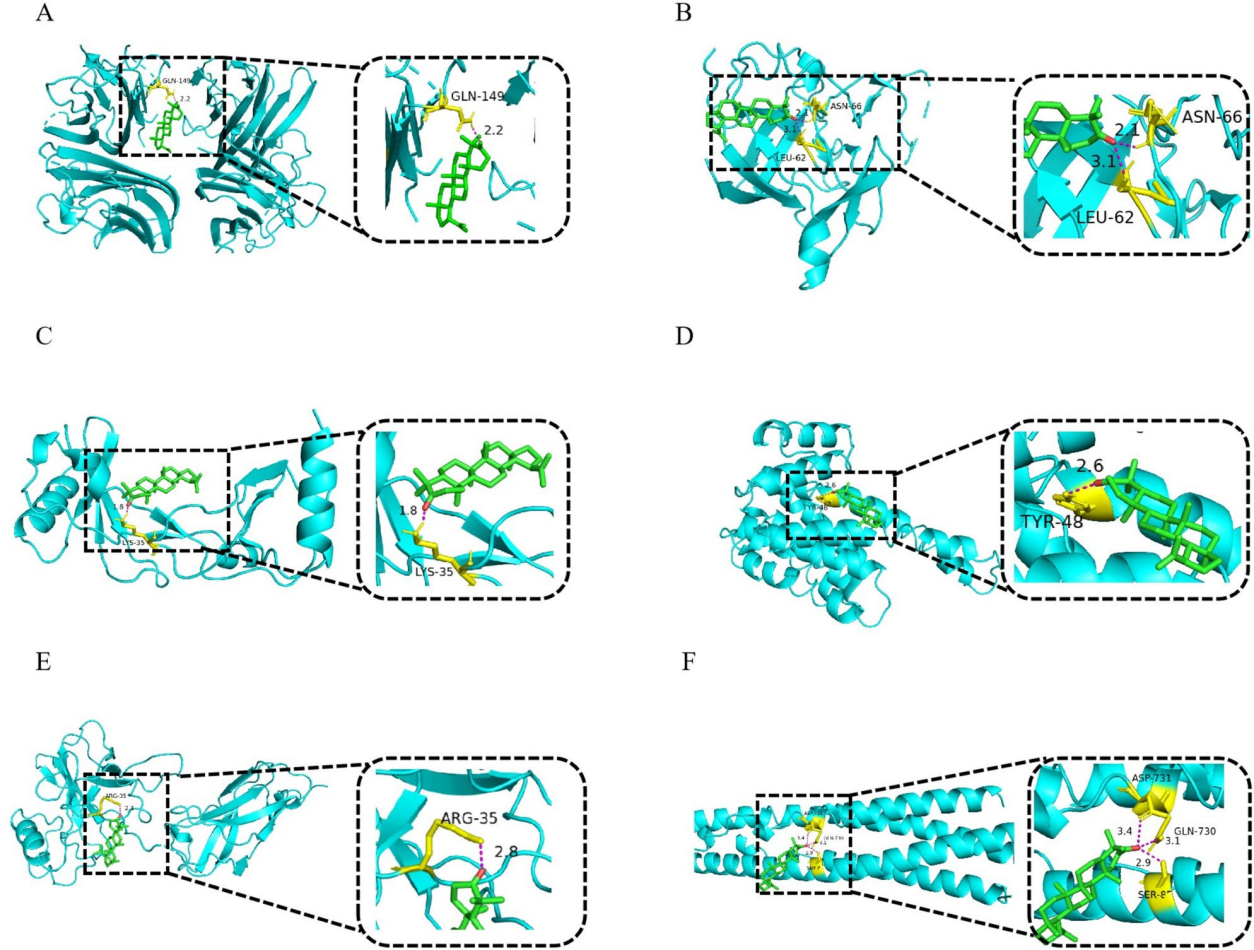
Molecular mechanisms of lupenone binding to the predicted protein target proteins (**A**) TNF-α (2AZ5), (**B**) IL-1β (3POK), (**C**) MCP-1 (1ODK), (**D**) IκBα (6Y1J), (**E**) P65 (1IKN) and (**F**) IKKβ (3BRV) are shown interacting with lupenone molecule. Green stick models represent lupenone, yellow represent residues in the binding sites, pink dashed lines represent hydrogen bonds, and the blue lines represent protein. Bond lengths are indicated next to the bonds.

Subsequently, we performed a molecular docking analysis using the in AutoDock software. Lupenone combined with six proteins (MCP-1, IL-1β, TNF-α, IKKβ, IκBα, and NF-κB p65) showed binding energies below − 5 kJ/mol (Table 2, Fig. 2A-F), and the smaller the binding energy, the greater the affifinity. The major interactions were hydrogen bonding and *π*-*π*-stacking, suggesting potentially favourable interactions between lupenone and its targets. Within the target proteins, certain amino acids (e.g., ARG, LYS, ASP, TYP, SER, etc.) can act as active centre essential groups for enzymes to catalyse chemical reactions.

**Table 2:** Information on the molecular docking results of the six significant proteins.

	PDB ID	Binding energy (kcal/mol)	PDB ID	Binding energy (kcal/mol)
Lupenone	2AZ5	− 8.71	6Y1J	− 6.45
	3POK	− 7.34	1IKN	− 6.21
	1ODK	− 6.56	3BRV	− 5.57

### Regulation of pro-inflammatory cytokine production by lupenone in high‐sugar-stimulated HBZY-1 cells

**Figure 3:**
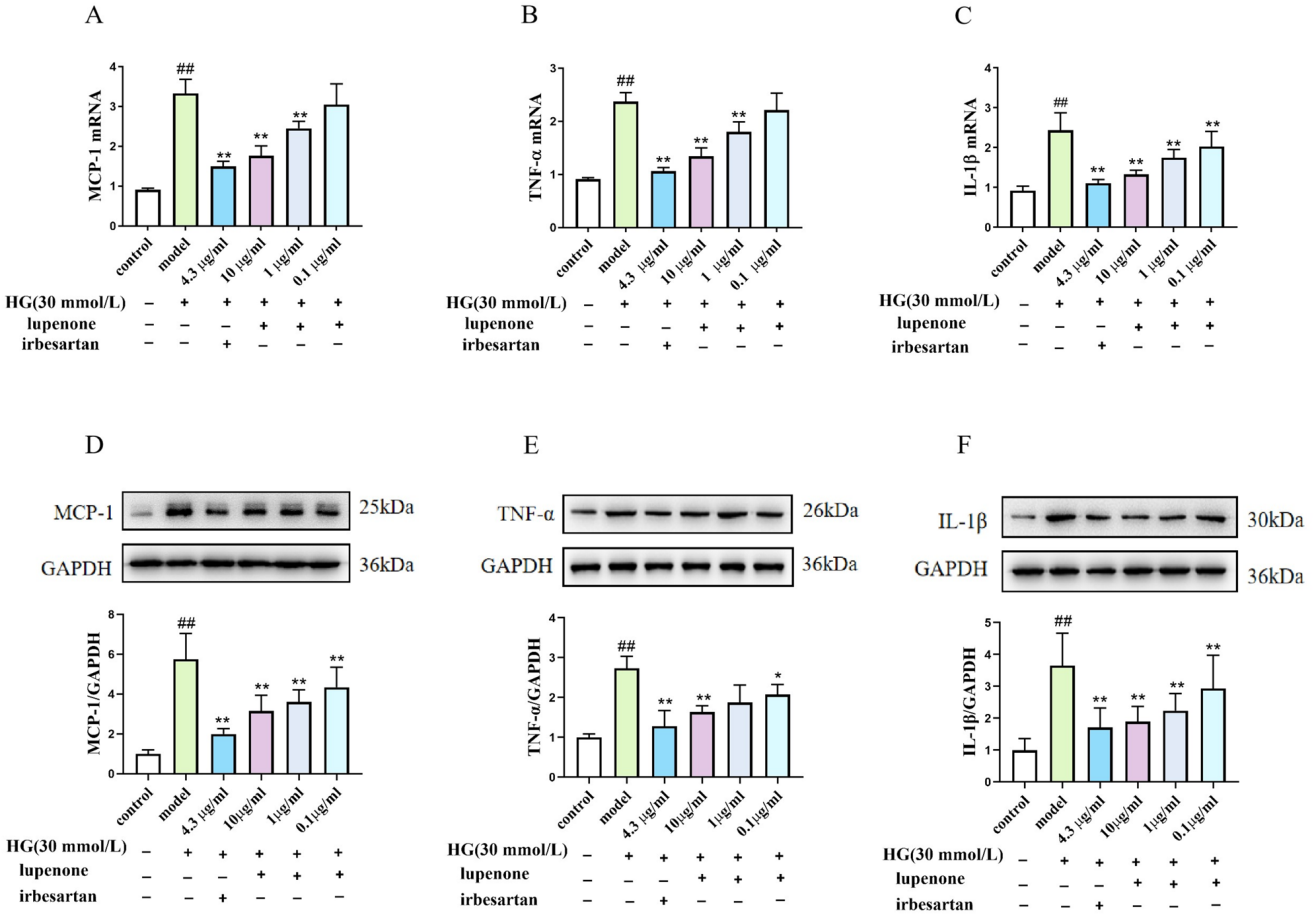
Effects of lupenone (10, 1, 0.1 μg/mL) and irbesartan (4.3 μg/mL) treatment (24 h) on MCP-1, IL-1β and TNF-α production in high-glucose-stimulated HBZY-1 cells. Results are presented as mean ± SD. Significance: ^##^*P* < 0.01 versus the control, **P < 0.01 versus the model.

Hyperglycaemic states result in the overexpression of renin receptors and their ligands, leading to the production of inflammatory factors, while anti-inflammatory compounds effectively attenuate the production of inflammatory factors in hyperglycaemia-induced DN^26,27^. The mRNA expression of MCP-1, TNF-α, and IL-1β was substantially elevated in HBZY-1 cells subjected to high-glucose stimulation (Fig. 3A-C). Both lupenone (10 μg/mL and 1 μg/mL) and the positive drug irbesartan (4.3 μg/mL) were able to downregulate the expression of these pro-inflammatory cytokines in HBZY-1 cells subjected to high-glucose stimulation (*P* < 0.01). Interestingly, the 0.1 μg/mL lupenone dose group showed markedly reduced IL-1β mRNA expression (*P* < 0.01), while MCP-1 and TNF-α mRNA expression levels tended to decrease, although the difference was not significantly (*P* > 0.05).

To further confirm the anti-inflammatory effect of lupenone on protein expression,,, western blot analysis was used to confirm the effect of lupenone on the expression of the pro-inflammatory cytokines. MCP-1, IL-1β, and TNF-α protein expression was elevated in HBZY-1 cells subjected to high-glucose stimulation (Fig. 3D-F) and reduced following treatment with irbesartan and lupenone under the same high-glucose conditions (*P* < 0.01). However, the 1 μg/mL lupenone dose group showed decreased expression levels of TNF-α, although the difference was not significant (*P* > 0.05).

### Effect of lupenone on the NF-κB pathway in HBZY-1 cells

**Figure 4:**
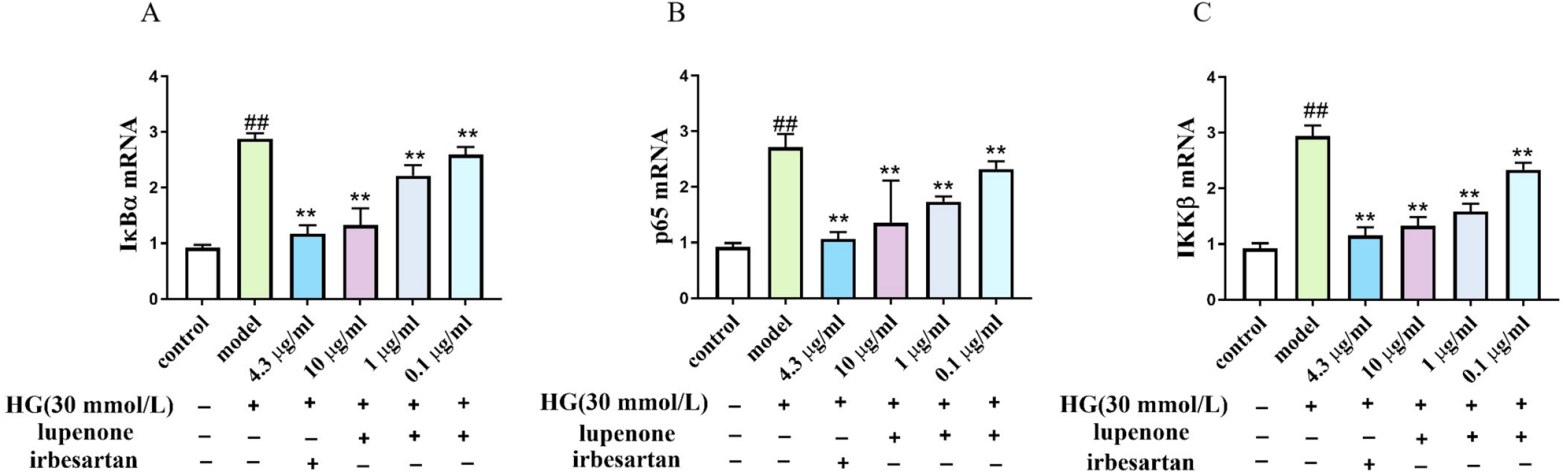
Effects of Lupenone (10, 1, 0.1 μg/mL) and irbesartan (4.3 μg/mL) treatment (24 h) on (**A**) IκBα, (B) p65, (**C**) IKKβ mRNA production in high-glucose-stimulated HBZY-1 cells. Results are presented as mean ± SD. Significance: ^##^*P* < 0.01 versus the control, **P < 0.01 versus the model.

**Figure 5:**
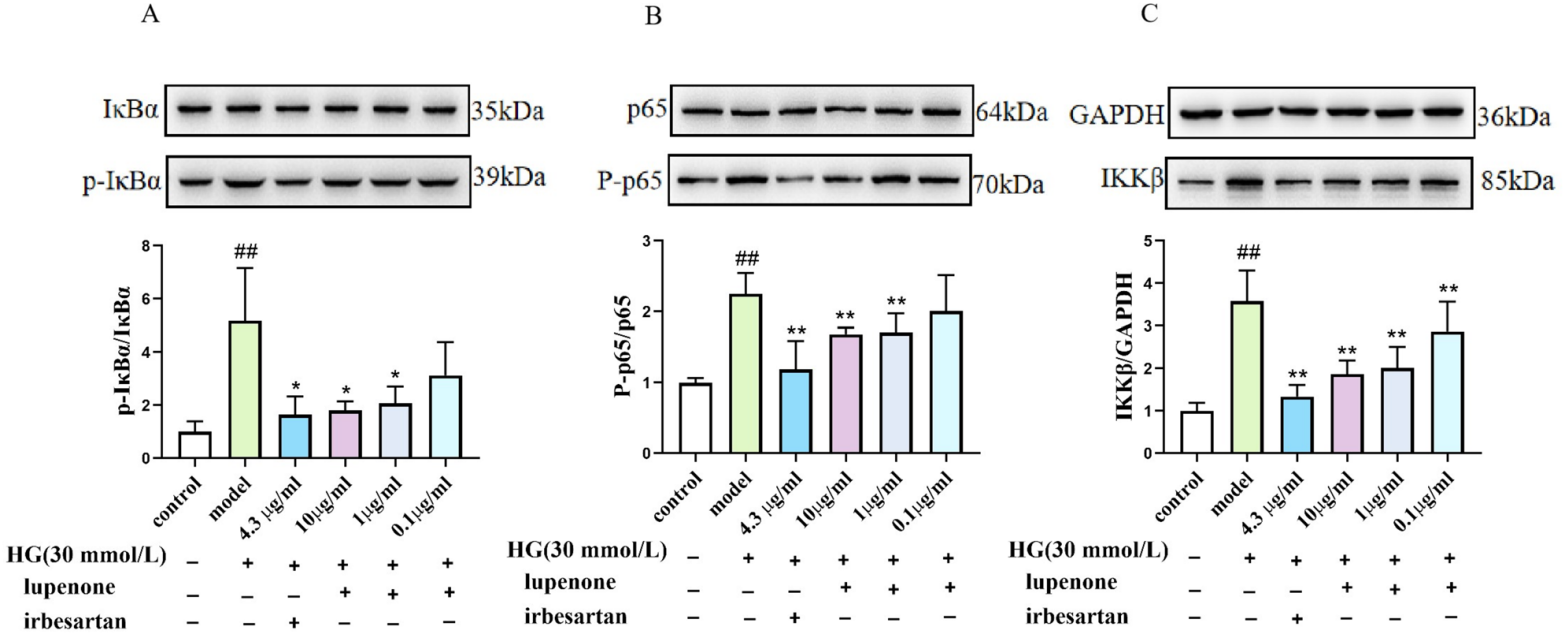
Effects of lupenone (10, 1, 0.1 μg/mL) and irbesartan (4.3 μg/mL) treatment (24 h) on the protein levels for (**A**) p-IκBα/IκBα, (**B**) p-p65/p65, and (**C**) IKKβ/GAPDH in high-glucose-stimulated HBZY-1 cells. Results are presented as mean ± SD. Significance: ^##^*P* < 0.01 versus the control, **P < 0.01 versus the model.

In addition, based on the inhibition of pro-inflammatory cytokine expression by lupenone, we further examined whether the effect of lupenone on inflammation in high‐glucose-induced HBZY-1 cells is associated with the NF-κB pathway. In HBZY-1 cells stimulated by high-glucose, both mRNA and protein expression of p65, IκBα, and IKKβ was significantly higher compared to that in the control group (*P* < 0.01, Fig. 4A-C). At the same time, we found that the gene expression of p65, IκBα, and IKKβ mRNA was significantly decreased in the irbesartan and lupenone (10, 1, 0.1 μg/ mL) dose groups compared with that in the model (*P* < 0.01). According to western blot results, The p-p65/p65 and IKKβ/GAPDH protein expression in the cell model was increased remarkably compared to that in the irbesartan (4.3 μg/mL) and lupenone (10 μg/mL, 1 μg/mL) dose groups after treatment. (*P* < 0.01, Fig. 5A-C). The p-IκBα/IκBα proteins expression in the model were increased remarkably than that in the irbesartan (4.3 μg/mL) and lupenone (10 μg/mL, 1 μg/mL) dose groups after treatment (*P* < 0.05, Fig. 5A-C). Furthermore, we also found that p-IκBα/IκBα and p-p65/p65 protein levels were reduced in the 0.1 μg/mL lupenone dose group, although the difference was considered not statistically remarkable (*P* > 0.05). This finding suggests that lupenone exerts an anti-inflammatory effect by inhibiting the expression of genes and proteins in the NF-κB pathway, preventing the phosphorylation of IKKβ and IκBα, and blocking the activation of NF-κB.

## Discussion

DN is the diabetic complication with the highest mortality rate, in the United States, 30% to 40% of patients with diabetes develop DN^28,29^. Recently, studies have shown that controlling inflammation can potentially improve DN^30^. Therefore, we recognised that effective improvement of the inflammatory response helps to control the development of DN. Compounds extracted from natural products that can improve inflammation in patients with DN, with relatively few side effects, have been the focus of drug development.

Lupenone was isolated from *Musa basjoo* Sied.et Zucc, *Musa nana* Lour, *Musa acuminata cv.* Mas (AA). Substantial evidence suggests that lupenone has multiple therapeutic roles in animals, such as anti-inflammatory effects and the prevention and the treatment of type 2 diabetes as well as the treatment of kidney damage^16–20^. However, the precise mechanisms in involved the inhibiting inflammatory response in DN remains uncertain. We believe that the anti-inflammatory properties of lupenone may mediate its protective effects against DN. Therefore, we cultured HBZY-1 cells in a high-glucose medium to generate a model that mimics the pathological causes of mesangial cell proliferation and the consequent renal dysfunction observed in the early stages of DN^31^.

Moreover, we used molecular docking technology to explore the molecular mechanism of lupenone action against DN. According to the molecular docking results, the molecular binding energies of lupenone with six target proteins were less than -5 kcal/mol, indicating a strong binding interaction. This indicated that the receptor and ligand can bind to each other under natural conditions, which indicates the presence of favourable interactions between lupenone and the six proteins. These results indicate that the six target proteins with a strong affinity for lupenone may play an important role in treating inflammation.

Natural compounds can ameliorate the inflammatory response and oxidative stress in DN with relatively few side effects^32,33^. To determine whether lupenone can relieve the inflammatory response in HBZY-1 cells cultured in a high-glucose medium, we evaluated the mRNA and protein expression of the pro-inflammatory factors MCP-1, TNF-α, and IL-1β. MCP-1 can also promote the migration and infiltration of inflammatory cells, such as monocytes, to the site of inflammation. Among them, M2b macrophages could produce TNF-α and IL-1β, which accelerate the inflammatory response^34,35^. In addition, evidence suggests that reducing the expression of MCP-1 could alleviate diabetes-related symptoms and relieve kidney impairment in early DN^36,37^. These findings suggest that inhibiting the expression of MCP-1 can play a role in improving DN. Furthermore, TNF-α and IL-1β are important inflammatory mediators that play key roles in the pathological processes of DN. Chen et al.^38^ confirmed that the IL-1β and TNF-α expression levels in kidney tissues was greatly increased compared to those in the control group, and their expression significantly decreased after drug treatment. IL-1β, a member of the cytokine family, is mainly released by endothelial cells, fibroblasts, and other cell types^39,40^. Notably, the pro-inflammatory factors IL-1β and TNF-α can activate the NF-κB signalling pathway, participate in the inflammatory process, and form an immune cascade^41,42^. In the present study, we found that lupenone could effectively alleviate the inflammatory response by inhibiting the proliferation of HBZY-1 cells and downregulating the expression of MCP-1, TNF-α, and IL-1β.

**Figure 6:**
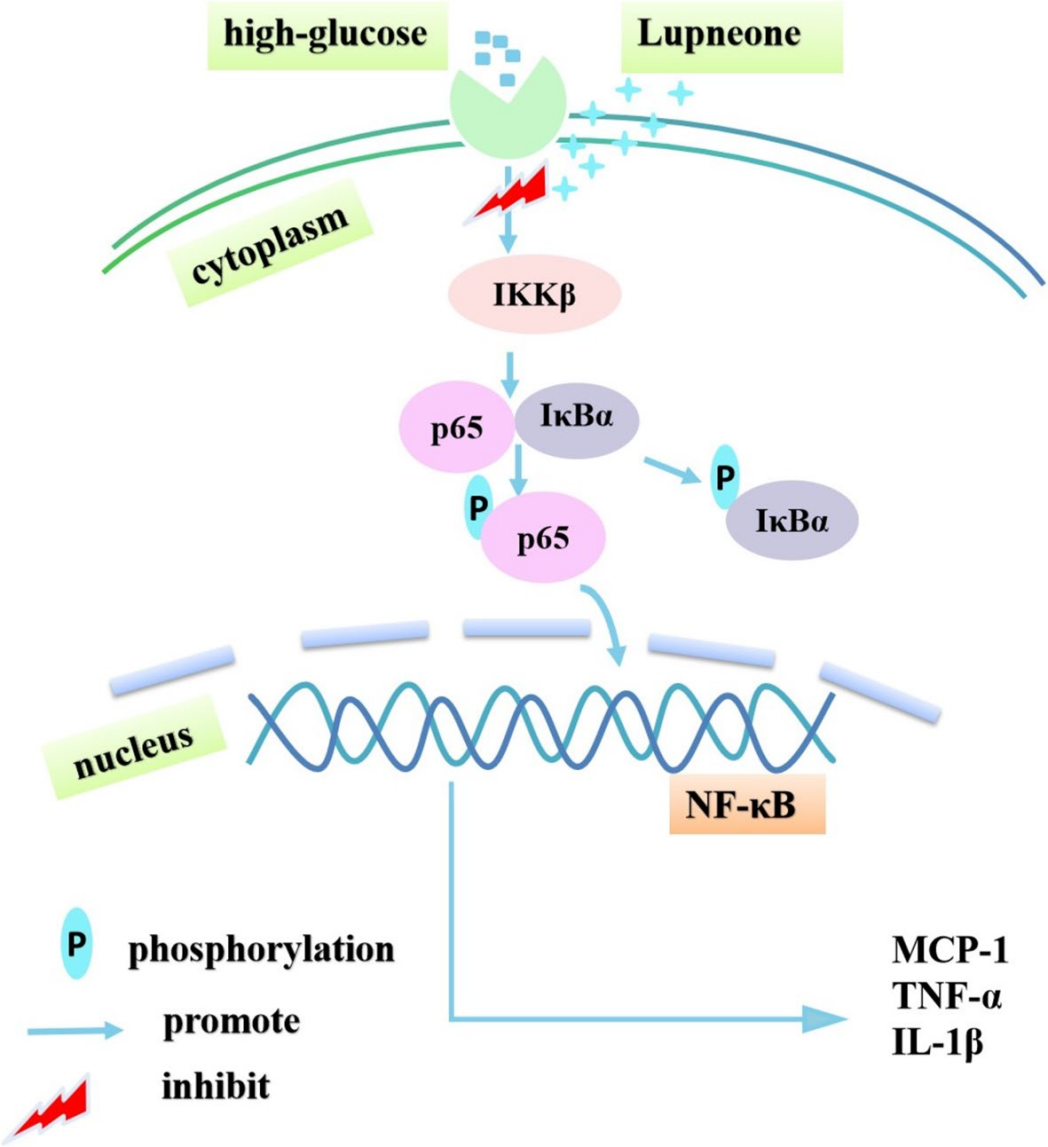
A schematic diagram showing proposed mechanism of action of lupenone in ameliorating the DN inflammatory response.

Next, we investigated whether lupenone regulates the NF-κB inflammatory pathway (Fig. 6). Our results indicated that treatment with lupenone downregulated p65, IκBα, and IKKβ mRNA and p-p65/p65, p-IκBα/IκBα, and IKKβ protein levels in HBZY-1 cells. In general, NF-κB is expressed in most tissue cells and is generally present in the cytoplasm in an inactive state. When cells are stimulated, NF-κB is translocated into the nucleus and binds to the NF-κB sites on target genes, thereby triggering the transcription of the target gene^43,44^. When the NF-κB signalling pathway is activated, it induces the transcription of a variety of inflammatory mediator genes, which causes chemotaxis inflammatory cells to infiltrate and accumulate at the inflammatory site, resulting in an inflammatory response^45^. A recent study found that activation of the NF-κB signalling pathway could induce MCP-1 synthesis and expression^46^. Zhong et al.^47^ also demonstrated that curcumin can exert anti-inflammatory effects by regulating the NF-κB pathway and p38 MAPK expression to inhibit the expression of MCP-1. Additionally, studies have shown that IL-1β and TNF-α can activate NF-κB,thereby, promoting the downstream pathway, leading to high expression of TNF-α and IL-1β, which then promotes the inflammatory response^48^. Research results indicate that NF-κB is the centre of the inflammatory response and is involved in the secretion of pro-inflammatory cytokines and the continuation and expansion of the inflammatory response^49,50^. Therefore, these results indicate that inhibition of NF-κB pathway activation is crucial for alleviating the inflammatory response. In agreement with the results of these reports, our findings confirmed that lupenone exerts anti-inflammatory effects through directly regulation of the NF-κB pathway, detected by molecular docking and in vitro cellular model experiments. Additionally,, our study used a high glucose-induced inflammation model of glomerular mesangial cells for the experiments, and the effect of lupenone on this cellular model has not been reported previously. Therefore, our research provides new evidence for the potential of lupenone in the treatment of diabetic nephropathy or nephrolithiasis.

## Materials and methods

### Reagents and antibodies

Lupenone was prepared at a purity of 98% by the Drug Analysis Laboratory of Guizhou University of Traditional Chinese Medicine. The chemical structure was confirmed using NMR spectroscopy and high-resolution mass spectrometry (Fig. 1A)^11^. Irbesartan was purchased from SANOFI Pharmaceuticals Co., Ltd. (Philadelphia, PA, USA). Antibodies against GAPDH, MCP-1, TNF-α, IL-1β, IκBα, IKKβ, NF-κB p65 and phospho-NF-κB p65 were purchased from Abcam Biotechnology Co. Ltd. (MA, USA). Antibodyies against phospho-IκBα were purchased from Affinity Biologicals, Inc. (Ancaster, ON, Canada). Dulbecco’s modified Eagle medium (DMEM), foetal bovine serum (FBS), and streptomycin penicillin were purchased from HyClone (Logan, Utah, USA).

### Cell culture

The HBZY-1 cell line was purchased from Wuhan Procell Life Science & Technology Co., Ltd., (Wuhan China) and was maintained in DMEM supplemented with 10% FBS, 100 U/mL penicillin, and 100 μg/mL streptomycin, at 37 °C with 5% CO_2._

Lupenone and irbesartan were dissolved in dimethyl sulfoxide (DMSO) (Sigma-Aldrich, St. Louis, MO, USA). The HBZY-1 cells were inoculated into 96-well plates. For the control group, cells were incubated without glucose medium,in the experimental groups, glucose (30 mmol/L) was added for stimulation, and then, cells were treated with lupenone (0, 0.1, 1 and 10 μg/mL) or irbesartan (4.3 μg/mL) , in a 5% CO_2_ incubator at 37 °C for 24 h. Cells between passage numbers 5 and 6 were used for subsequent experiments.

### Effects of lupenone on the viability of HBZY-1 cells in the absence of glucose

HBZY-1 cells were inoculated into 96-well plates and treated with lupenone at 0 ng/mL, 1 ng/mL- 100 μg/mL in a CO_2_ atmosphere at 37 °C for 24 h. Cell viability was measured by MTT method^51^. Finally, the optical density (OD) values were measured using a microplate reader (Thermo Fisher Scientific) at 570 nm.

### Effects of lupenone on the viability of HBZY1 cells in the presence of glucose

Subsequently, the cells were randomly divided into eight groups. Except for the control, 30 mmol/L glucose was added and cells were treated with lupenone (0 μg/mL, 1 ng/mL-100 μg/mL) for 24 h, then the cell proliferation rate of each group was determined using the MTT method. The OD values at 570 nm were detected using a microplate Reader.

### RT-PCR

Total RNA was extracted from cultured cells using TRIzol (Takara, Kyoto, Japan), according to the manufacturer's instructions. Complementary DNA (cDNA) was synthesized using the TAKARA PrimeScript™RT reagent Kit with gDNA Eraser (Takara, Kyoto, Japan), as per the protocol. The cDNA samples were individually configured for amplification using RT-PCR. The target mRNA levels were normalized against GAPDH mRNA relative to the control and were calculated using the 2^−∆∆CT^ method. The primer sequences used are shown in Table 3.

**Table 3:** Detailed sequences of the primers used in the RT-PCR experiments.

Gene	Forward primer (5′–3′)	Reverse primer (5′–3′)
GAPDH	GGGAAACCCATCACCATCTT	CCAGTAGACTCCACGACATACT
MCP-1	GTCTCAGCCAGATGCAGTTAAT	CTGCTGGTGATTCTCTTGTAGTT
TNF-α	ACCTTATCTACTCCCAGGTTCT	GGCTGACTTTCTCCTGGTATG
IL-1β	TCCCTGAACTCAACTGTGAAATA	GGCTTGGAAGCAATCCTTAATC
p65	ACCTGATGCAGAACGGTAAG	GCTGAAGGACTCGTTGTAGTAG
IkBα	AGTAACCTACCAGGGCTACTC	ATAGCTCTCCTCATCCTCACTC
IKKβ	AGAAAGTGCGGGTGATTTACT	CCTCACCACCTCTTCTACTTTG

### Western blot

The protein-level expression of MCP-1, IL-1β, TNF-α, p65, p-p65, IκBα, p-IκBα and IKKβ was determined using western blotting. Following different treatments, total protein was extracted from HBZY-1 cells and isolated using radio-immunoprecipitation assay (RIPA) buffer, and the lysate was centrifuged for 10 min at 4 ℃and 12,000 rpm). Then we collected the supernatant and extracted the proteins and a bicinchoninic acid (BCA) protein kit was used to determine the proteins concentration. Finally, the protein bands were visualised with a chemiluminescence reagent, Tanon ECL, and a multifunctional imaging system (Tanon 5200) was used for imaging (Shanghai, China).

### Molecular docking

Molecular docking is routinely used for understanding protein–receptor interaction between complexes^52^. In order to better understand the interactions between lupenone and the six target proteins (MCP-1, IL-1β, TNF-α, IKKβ, IκBα, and NF-κB p65), the AutoDockTools-1.5.7 software was used for molecular docking by fitting lupenone into the active site of the six proteins. We downloaded the 3D structures of target proteins from the PDB database (https://www1.rcsb.org/structure/6d3o). Furthermore, the lupenone structure was obtained from the Traditional Chinese Medicine Systems Pharmacology Database (https://tcmsp-e.com/tcmsp.php) and was saved in MOL2 format^53^. All the obtained 3D structures were imported into PyMOL software for dehydration and then into AutoDock for hydrogenation. The lupenone structure was imported into AutoDock tool as the docking ligand and saved in PDBQT format. All flexible keys were set to ‘rotatable’ by default. AutoDock tools were used for docking, and PyMOL was used to visualize the docking result.

### Statistical analysis

All data were analysed using SPSS 26.0 (SPSS Inc., Chicago, IL, USA). Normality was estimated using the Shapiro–Wilk test. Analysis of variance (ANOVA) was used to assess the statistical significance of the results, and *P* < 0.05 was considered statistically significant.

## Conclusions

In conclusion, our study suggest that the therapeutic effects of lupenone in DN may be mediated through downregulation of the NF-κB pathway, and thus lupenone exerts an anti-inflammatory role. Hence, these findings provide important experimental evidence for the development of lupenone as a potential drug for DN prevention.

### Supplementary Information

Supplementary Information.

## Data Availability

The datasets generated during and/or analyzed during the current study are available from the corresponding author upon reasonable request.
